# Swimbladder function and the spawning migration of the European eel *Anguilla anguilla*

**DOI:** 10.3389/fphys.2014.00486

**Published:** 2015-01-05

**Authors:** Bernd Pelster

**Affiliations:** ^1^Institute for Zoology, University of InnsbruckInnsbruck, Austria; ^2^Center for Molecular Biosciences, University of InnsbruckInnsbruck, Austria

**Keywords:** swimbladder function, *rete mirabile*, gas gland cells, European eel, buoyancy, spawning migration

## Abstract

The spawning migration of the European eel is an extensive journey over 5000 to 7000 km from the European coast to the Sargasso Sea. Eels do not feed during this journey and on-board fuels must be sufficient to support the journey of 3.5 to 6 month, as well as sexual maturation and the spawning activity. Swimming of eels appears to be quite energy efficient compared to other fish species, and elevated hydrostatic pressure has been shown to even reduce the costs of transport. Recent studies revealed, however, that during traveling eels perform extensive diurnal migrations and swim at a depth of about 100–300 m at night time, but go down to 600–1000 m at day time. At a depth of 200 m eels are exposed to a hydrostatic pressure of 21 atmospheres (2.13 MPa), while at 800 m hydrostatic pressure increases to 81 atmospheres (8.21 MPa). Accordingly, without any compensation at a depth of 800 m swimbladder volume will be reduced to about 25% of the volume established with neutral buoyancy at 200 m. Consequently, these diurnal changes in depth must be taken into consideration for a calculation of the energy requirements of the spawning migration. Without compensation a compression of the swimbladder will result in a status of negative buoyancy, which makes swimming more costly. Trying to keep the status of neutral buoyancy during descent by gas secretion into the swimbladder in turn requires metabolic activity to enhance swimbladder perfusion and for acid production of the gas gland cells to stimulate gas secretion. During ascent gas is passively removed from the swimbladder in the resorbing section and in the blood transported to the gills, where it is lost into the water. Accordingly, the swimbladder appears to be a crucial organ for the spawning migration. It can be assumed that an impairment of swimbladder function for example due to an infection with the nematode *Anguillicola crassus* significantly threatens the success of the spawning migration.

## Introduction

For centuries, the European eel (*Anguilla anguilla*, L) has been an important target species for fishers all over Europe (Tesch, 1999). However, since the 1980s, the stock has been in a steep decline and alarmingly low recruitment numbers are documented in virtually every time series available as well as reflected in landing numbers all over Europe (Dekker, 2003; ICES Advisory Committee, 2013). Nowadays, the European eel stock is considered to be out of safe biological limits and the species is listed in Appendices I–III of the Convention on International Trade in Endangered Species (CITES, 2013).

Reasons currently discussed for this decline are diverse and include exploitation, the loss of habitats, increased mortality due to river obstacles (ICES, 2006) and possible climatic and oceanic changes such as increasing water temperatures in the spawning area, unfavorable wind-driven currents or a shifting of isotherms (Knights, 2003; Friedland et al., 2007; Bonhommeau et al., 2008; Durif et al., 2011; Kettle et al., 2011; Baltazar-Soares et al., 2014). Beside these, habitat and spawner quality are considered major factors influencing recruitment success (Belpaire et al., 2009; Geeraerts and Belpaire, 2010; Clevestam et al., 2011). Due to its complex life cycle *A. anguilla* is specifically vulnerable to environmental changes that potentially impair its ability for long-distance migration, a prerequisite for successful reproduction. To reach its spawning area in the Sargasso Sea (Schmidt, 1923), mature *A. anguilla* have to migrate distances between 5000 and 7000 km, known as the longest spawning migration within the genus *Anguilla* (Aoyama, 2009) and estimated to last between 3.5 and 6 months of continuous swimming (Palstra and van den Thillart, 2010). Animal condition and swimming performance can be severely impaired by a variety of environmental factors like contaminant loads (van Ginneken et al., 2009; Geeraerts and Belpaire, 2010), infection with the introduced swimbladder nematode *Anguillicola crassus* (Kirk, 2003; Palstra et al., 2007; Clevestam et al., 2011) and a lack of energy resources (Svedäng and Wickström, 1997).

Recent attempts to track the spawning migration of the European eel using pop-up satellite archival transmitter tags suggested that the swimbladder as a buoyancy organ may be of special importance during the migration. At night time they travel in the upper water level at a depth of about 100 to 300 m, while at daytime they prefer deeper water layers between 500 and 700 m, and may even go down to 1000 m (Aarestrup et al., 2009). Although these diurnal migrations typically are not performed in neutral buoyancy at all water levels (Pelster, 1997, 2009, 2013; Sebert et al., 2009b), this observation clearly stresses that a functioning swimbladder is essential and probably indispensable for a successful completion of the spawning migration. During this time eels do not feed and the alimentary canal atrophies (Tesch, 1999). Accordingly, the whole migration culminating in sexual maturity and reproduction must be fueled by on board stores. Several studies tried to obtain an estimate of the energetics of this journey and to relate it to the onboard stores at the onset of the journey (Van den Thillart et al., 2009). Eels appear to have developed a very efficient way of swimming; the cost of transport has been shown to be much lower than in trout, for example. Nevertheless, remaining at a certain water depth is costly for a fish with an overall body density much higher than sea water density, and daily migrations over a depth range of several hundred meters certainly require appropriate adjustments. A functioning swimbladder in this situation significantly contributes to energy saving (Alexander, 1972, 1990; Pelster, 2009, 2013). A reduced swimbladder function in turn will cause an increase in negative buoyancy and in the cost of transport. This most likely will reduce the chances to reach the spawning sites in the Sargasso Sea (Van den Thillart et al., 2009) and thus contribute to a decline in the population of the European eel. Starting with a short description of swimbladder structure and function this paper therefore analyzes how the swimbladder can contribute to vertical migrations and to a successful spawning migration of the eel. This includes a consideration of the possible consequences for the energy requirements for the migration and the impact of an infection of the swimbladder with the nematode *Anguillicola crassus*, which within less than a decade was spread all over Europe. Because at depth oxygen is assumed to be the main swimbladder gas swimbladder tissue is exposed to tremendously high oxygen partial pressures. Therefore, the question how the swimbladder tissue is protected against the formation of reactive oxygen species (ROS) will also be discussed.

## Swimbladder structure and function

The swimbladder of the eel has extensively been used as a model for swimbladder function in fish because blood is supplied to the eel swimbladder via a bipolar countercurrent system, the so-called “Wundernetz” or *rete mirabile*, which allows for a separate analysis of the functioning of the countercurrent system and of the gas gland cells, which in the eel represent the swimbladder epithelium and are responsible for the initiation of gas secretion (Pelster, 1997, 2009).

**Figure 1:**
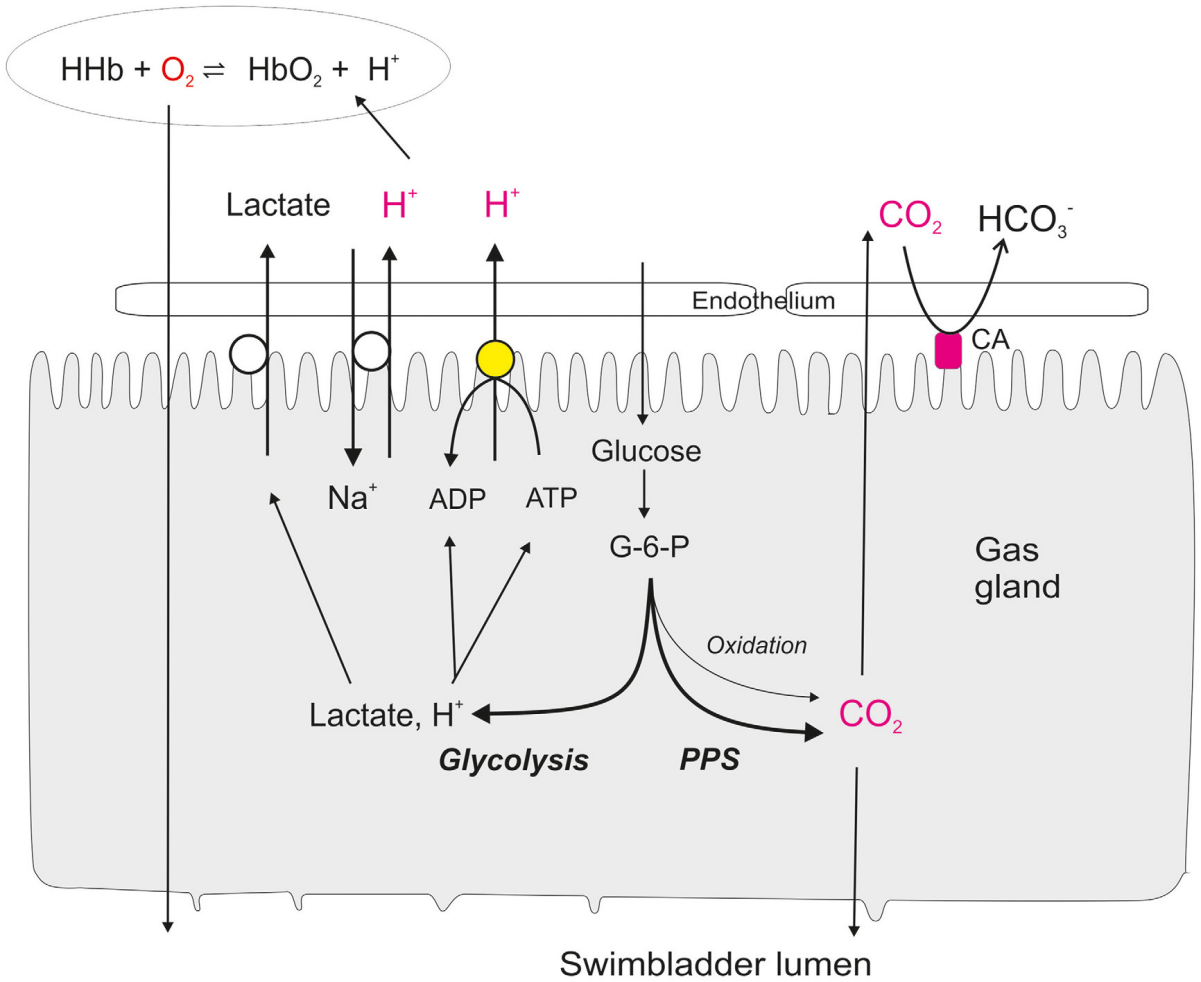
**Present concept of glucose metabolism and the secretory activity of swimbladder gas gland tissue**. Glucose is taken up from the blood and mainly converted into lactate, in spite of the fact that gas gland tissue typically is exposed to high oxygen partial pressures. A fraction of the glucose is converted to CO_2_ in the pentose phosphate shunt (*PPS*). Only a very small fraction of the glucose is oxidized by aerobic metabolism. The CO_2_ produced in the cell diffuses down the partial pressure gradient into the swimbladder lumen as well as into the blood. A membrane bound carbonic anhydrase (CA) rapidly establishes the equilibrium between CO_2_ and HCO^−^_3_ in the extracellular space and in the blood. Protons are secreted into the blood via a proton ATPase and sodium-proton exchange (NHE). The acidification of the erythrocytes switches on the Root effect and thus reduces the oxygen carrying capacity of the hemoglobin. Oxygen is released from the hemoglobin and diffuses down the partial pressure gradient through the gas gland cells into the swimbladder lumen. Lactate is released into the blood and contributes to the salting out effect.

Gas molecules simply diffuse along partial pressure gradients from the blood into the swimbladder, and gas secretion therefore is a passive phenomenon. Thus, an initial increase in gas partial pressure is required, the so-called single concentrating effect (Kuhn et al., 1963), which is achieved by a reduction in physical solubility of gases or a decrease in gas carrying capacity of the blood (Root effect). This single concentrating effect is achieved by metabolic activity of the gas gland cells, which produce and secrete lactic acid, even though they are exposed to very high oxygen partial pressures. In the eel swimbladder about 80% of the glucose removed from the blood is converted to lactic acid, and the release of lactate and protons by gas gland cells significantly acidifies the blood (Pelster, 1995). They also produce and release CO_2_, mainly generated in the pentose phosphate shunt, i.e., without concomitant consumption of oxygen (Walsh and Milligan, 1993; Pelster et al., 1994). Due to the production of CO_2_ in the pentose phosphate shunt a high PCO_2_ has to be expected in gas gland cells, driving the outward diffusion of CO_2_ into the blood, but also into the swimbladder (Figure 1). This contributes to the acidification of blood during passage of the gas gland cells. Accordingly, in the European eel *Anguilla anguilla* blood returning to the *rete mirabile* is significantly acidified after passing the metabolically active gas gland cells (Steen, 1963; Kobayashi et al., 1990b), and this acidification reduces the hemoglobin oxygen carrying capacity via the Root effect (Root, 1931; Pelster and Randall, 1998; Pelster, 2001). Figure 1 summarizes our current knowledge about the metabolism of gas gland cells and the various mechanisms contributing to the release of protons, lactate and CO_2_ from the cells into the blood.

These considerations show that during passage of the gas gland cells the metabolic activity of these cells induces an initial increase in gas partial pressure of all gases in the blood. Depending on the rate of acidification and on the hemoglobin concentration this effect may be very large for oxygen (Pelster, 2001), while for inert gases including nitrogen the salting out effect induced increase in gas partial pressure probably is only quite small due to the comparatively small increase in total solute concentration (Pelster et al., 1988). For PCO_2_ also a significant increase can be expected, depending on the activity of the pentose phosphate shunt (Steen, 1963; Kobayashi et al., 1990b; Pelster, 2013).

**Figure 2:**
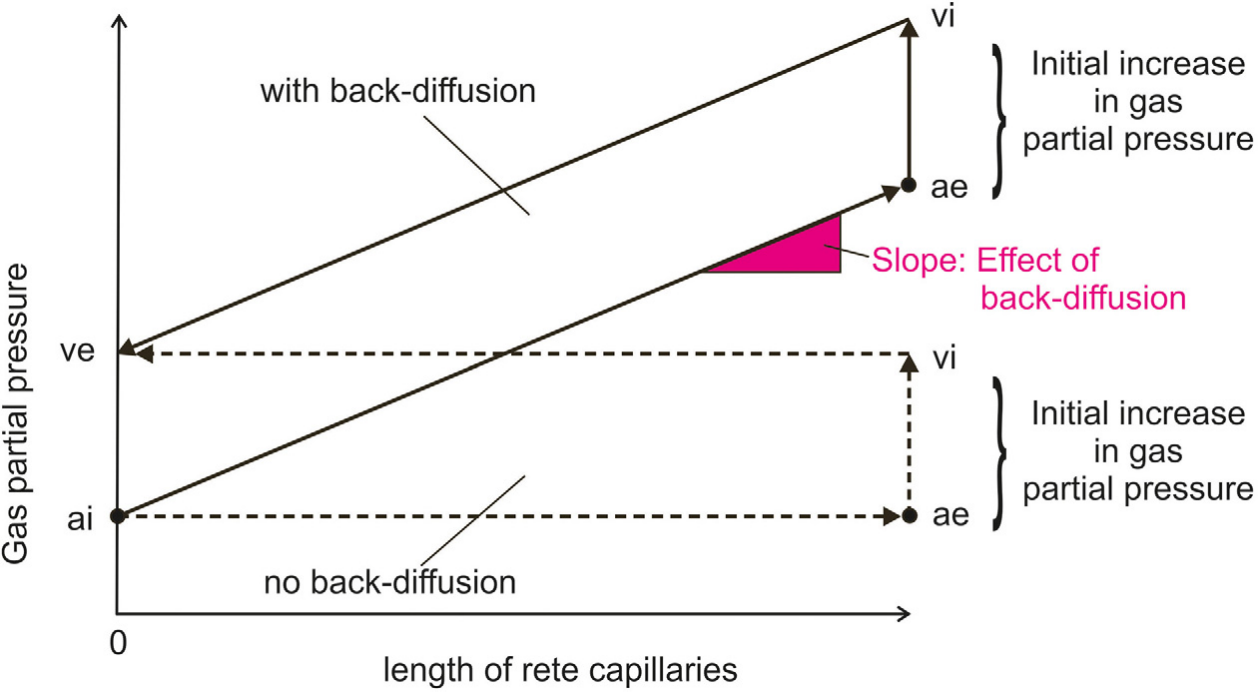
**Schema of inert gas partial pressure changes in the *rete mirabile* with and without back-diffusion from the venous to the arterial side of the *rete***. The situation is more complex for oxygen and CO_2_, because pH dependent changes in chemical binding will affect partial pressures, and the *rete* is not only permeable to gases, but also to small metabolites. The initial increase in gas partial pressure is brought about by the secretory activity (acid, lactate) of gas gland cells. ai, arterial influx of the *rete mirabile*; ae, arterial efflux; vi, venous influx; ve, venous efflux.

The increase in gas partial pressure in blood and the increase in PCO_2_ in gas gland cells due to metabolic production will generate a pressure head for the diffusion of gas molecules into the swimbladder, but also lay ground for the second step in gas deposition, the multiplication of this initial increase in gas partial pressures by back-diffusion of gas molecules from the venous to the arterial side of the countercurrent system of the swimbladder, the *rete mirabile*. This results in the multiplication of the single concentrating effect in a countercurrent system, so that very high gas partial pressures can be achieved (Kuhn et al., 1963; Kobayashi et al., 1990a; Pelster, 2001, 2009, 2013). Figure 2 illustrates that in addition to the magnitude of the single concentrating effect (the initial increase in gas partial pressure), the rate of back-diffusion in the countercurrent system, which is dependent on the permeability of membranes and perhaps the presence of special transport proteins, and the length of the *rete* capillaries determine the magnitude of the partial pressure that can be achieved by countercurrent concentration. The initial increase in gas partial pressure measured in the swimbladder is largest for oxygen and therefore oxygen makes up the largest fraction in newly secreted gas, followed by CO_2_.

The histological changes of swimbladder tissue of different eel species observed during the preparation of the spawning migration support the conclusion that the swimbladder is of major importance for the journey of the European eel to the Sargasso Sea. Silvering includes a significant enlargement of the *retia mirabilia*, indicating an improvement of the countercurrent concentrating ability. In addition, vascularization and swimbladder wall thickness increase as well as guanine deposition in the eel swimbladder wall, which decreases its gas permeability and thus reduces diffusional loss of gas through the swimbladder wall (Kleckner, 1980a,b; Yamada et al., 2001). In the American eel *Anguilla rostrata* a 5-fold increase in the rate of gas deposition has been recorded in silver eels (Kleckner, 1980a). It therefore is assumed that this maturation is connected to a significant improvement in swimbladder function (Sebert et al., 2009b; Righton et al., 2012).

## The swimbladder during vertical migrations

Vertical migrations are observed for several fish species with a swimbladder (Marshall, 1972; Vent and Pickwell, 1977; Gee, 1983; Kalish et al., 1986; Neilson and Perry, 1990). Myctophids, for example, are well-known for their daily migrations between the epipelagic zone at night and a depth of 300–700 m during daytime (Watanabe et al., 2001), and also cod has been shown to travel frequently between 50 and 200 m, although the movements do not appear as regular as in Myctophids (Strand et al., 2005). The possible importance of the swimbladder for vertical migrations therefore has been questioned repeatedly and model calculations have been used to predict its possible function. It must be pointed out, however, that experimental data on swimbladder function during vertical migrations are scarce and existing models are based on a number of assumptions, which have not yet been verified. Gas secretion has been measured under atmospheric pressure and silvering has been shown to improve secretion (Kleckner, 1980a), but how much gas can effectively be secreted at a depth of several hundred meters is unclear. Gas pressure in the swimbladder is higher than the pressure in the surrounding water, so that gas must be lost from the swimbladder simply by diffusion along the partial pressure gradient. Because the partial pressure difference between the swimbladder and the surrounding water increases with depth, this diffusional loss increases with depth. It is known that guanine incrustation for example significantly reduces the gas permeability of the swimbladder wall as compared to other tissues, but it remains unclear how permeable the swimbladder wall is when fish dwell at a depth of several hundred meters. Similarly, gas in contact with an oval or in the resorbing part of the swimbladder will have a much higher partial pressure than the gas in the blood or the surrounding water. Accordingly, gas will be absorbed by the blood along the partial pressure gradient, transported to the gills in the venous circulation and lost into the water. But gas absorption has not been measured at depth and it is not known how much gas can effectively be resorbed. Keeping these uncertainties in mind we still can draw a reasonable picture about the possible role of the swimbladder during vertical migrations, and thus during the spawning migration of the eel.

The increase in hydrostatic pressure with increasing depth compresses the swimbladder, and in a typical teleost the swimbladder wall is not restrained by surrounding tissue. Therefore, swimbladder volume changes with changing hydrostatic pressure according to Boyle's law, except for Cyprinidae, which have rather inextensible walls, probably connected to the role of the swimbladder as an auditory organ (Alexander, 1966, 1972). It is generally assumed that fish are near neutrally buoyant at the upper level of their migration, and negatively buoyant at the lower level (Kanwisher and Ebeling, 1957; Alexander, 1972; Pelster, 2009; Sebert et al., 2009b). There are several good reasons for this. Gas deposition rates recorded so far reveal that gas deposition is a slow process. Bluefish (*Pomatomus saltatrix)* appears to be the fish that deposits gas fastest with about 4 h for filling the swimbladder, but usually it takes about 1 or 2 days for a complete swimbladder filling (Alexander, 1966). Furthermore, if fish with a gas-filled swimbladder were neutrally buoyant at the lower level of their migration the swimbladder would expand during ascent and the fish would rapidly become positively buoyant. Although gas reabsorption is faster than gas deposition, it is limited by blood flow to the resorbing section of the swimbladder and gas transport capacity of the blood and therefore too slow to compensate for the increase in swimbladder volume occurring during a rapid raise over a few hundred meters depth, which often is completed within 1 or 2 h (Strand et al., 2005). American yellow perch (*Perca flavescens)* is able to comfortably compensate a reduction in pressure of 16%, but loses control at a pressure reduction of 32% (Jones, 1952). Accordingly, if fish were neutrally buoyant at the lower level of their distribution range they would be endangered to lose control during a rapid ascent due to the expansion of the swimbladder and the concomitant decrease in overall density.

**Figure 3:**
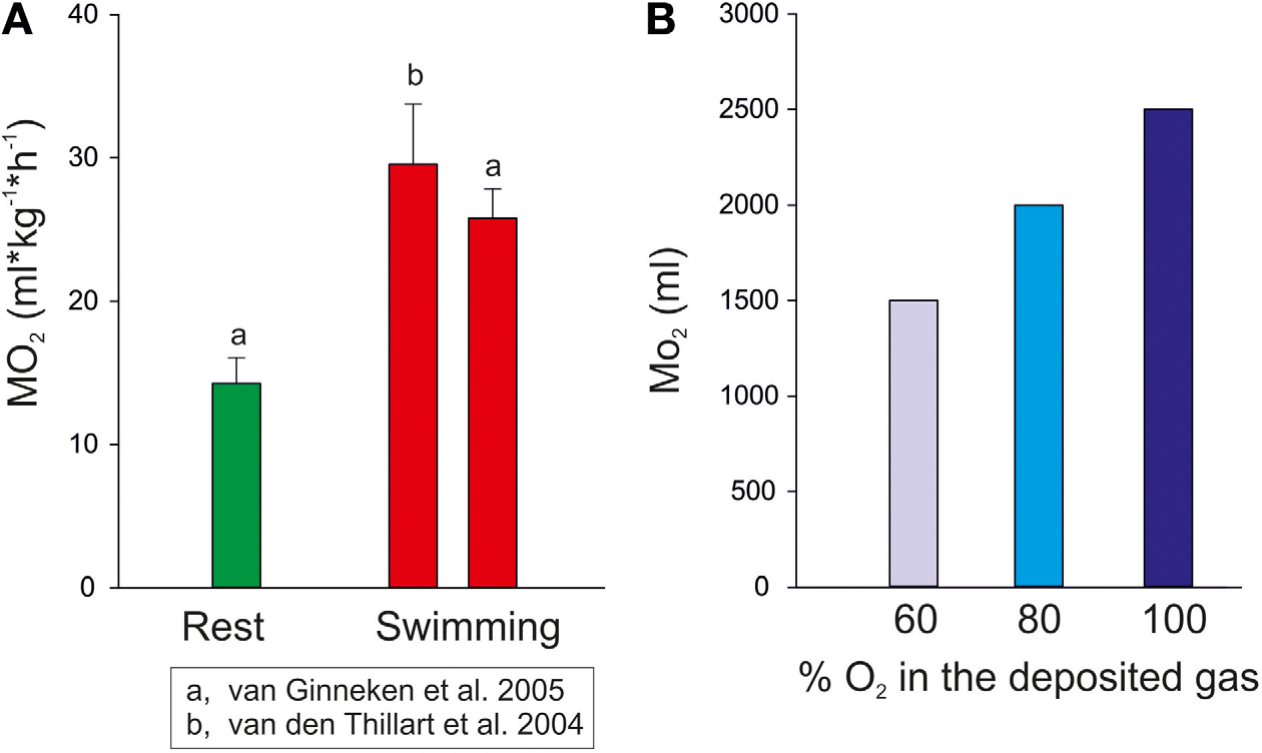
**(A)** Oxygen consumption of European eels measured at rest and during aerobic exercise (mean ± S.D.) in a swim tunnel (data taken from Van den Thillart et al., 2004 and from van Ginneken et al., 2005). **(B)** The amount of oxygen required for 1 kg eel to keep the swimbladder volume constant at 50 ml when descending from 300 to 800 m.

Considering the diurnal migration of the European eel we can assume an average change in depth between 300 m at nighttime and 800 m during the day (Aarestrup et al., 2009). If we take a 1 kg fish a swimbladder volume of about 50 ml is required to achieve neutral buoyancy (Alexander, 1966, 1971). Hydrostatic pressure at 300 m is 31 atm, therefore a swimbladder volume of 50 ml at this pressure would be equivalent to a volume of 1550 ml at the pressure of 1 atm at the water surface. If the eel then descends to 800 m hydrostatic pressure increases to 81 atm. To remain neutrally buoyant the fish must retain the volume of 50 ml, and according to Boyle's law 50 ml under a pressure of 81 atm would be equivalent to a volume of 4.050 ml at the water surface. Accordingly, the eel would have to secrete 2.500 ml of gas within the 1 or 2 h descent from 300 to 800 m, assuming constant temperature. Figure 3 shows the amount of oxygen that would be required to keep the volume of the swimbladder constant during the descent in comparison to the oxygen consumption measured in resting and swimming eel. For these calculations it is assumed that at depth the newly secreted gas is almost exclusively oxygen (Alexander, 1966, 1972). Alternatively, a more conservative value of about 60% oxygen in newly secreted gas has been used, which was measured under lab conditions in the European eel (Pelster and Scheid, 1992). Taking a swimbladder perfusion of about 1 ml^*^min^−1^ and the oxygen carrying capacity of the blood with a hemoglobin concentration of about 5–6 mmol^*^L^−1^ (Kobayashi et al., 1990b; Pelster and Scheid, 1992) it is obvious that the amount of oxygen required to keep the volume of the swimbladder constant during the descent is orders of magnitude greater than the normal oxygen consumption and orders of magnitude greater than could be supplied by the circulatory system.

Newly secreted gas often contains considerable amounts of CO_2_ (Meesters and Nagel, 1935; Copeland, 1952; Wittenberg et al., 1964), and in the European eel CO_2_ may make up to 25% of the newly secreted gas (Kobayashi et al., 1990b; Pelster and Scheid, 1992). It is expected that the contribution of CO_2_ is reduced at depth, but if the swimbladder volume is to remain constant during vertical migrations gas deposition must be reduced for a couple of hours when swimming constantly at the upper level in order to avoid floating, and then switched on again during the next descent. Gas secretion is initiated by an acidification of the blood during passage of the gas gland cells, and this acidification is achieved by production of lactic acid from glycolysis, and of CO_2_, mainly generated in the pentose phosphate shunt (Steen, 1963; Walsh and Milligan, 1993; Pelster et al., 1994; Pelster, 2001). Thus, the initiation of gas secretion does consume glucose for the production of lactic acid and of CO_2_, and the rate of gas secretion is related to the glucose consumption by the swimbladder tissue. Under atmospheric pressure about 0.118 mmol^*^h^−1^ (21.2 mg^*^h^−1^) glucose are required for the secretion of 1 ml^*^h^−1^ of gas in the European eel (Pelster and Scheid, 1993). In a long-term experiment van Ginneken et al. (2005) simulated the spawning migration of the European eel to the Sargasso Sea and observed a decrease in dry matter of 84.3 g^*^kg^−1^ after 6 month of swimming, compared to 42.7 g^*^kg^−1^ after 6 month of resting and fasting. Eels used mostly fat for swimming and dry mass carbohydrate content was below 1% at the start and at the end of the 6 month experiment (van Ginneken et al., 2005). From these data we can calculate that a 1 kg eel would not consume more than about 1 to 2 g of carbohydrate for the journey. Accordingly, the amount of carbohydrate consumed in this 6 month swimming experiment but also the onboard carbohydrate stores would not be sufficient to supply the swimbladder with enough glucose to support the required gas deposition. Based on these considerations it appears impossible that the eel will be able to use the swimbladder to retain neutral buoyancy during the observed vertical migrations, and we have to expect that it is near neutrally buoyant only during night time, when it swims in the upper water layers. When descending into deeper layers the eel will become negatively buoyant, and this deficit in buoyancy must be compensated by hydrodynamic lift, i.e., by swimming activity, which in turn requires energy.

## Energetics

The energetics of buoyancy and of swimming activity has been extensively studied by Alexander (1966, 1971, 1990). If whole body density of a fish is equal to the density of water the fish has no weight in water, it is neutrally buoyant. If it is denser than water, it is negatively buoyant and will tend to sink. Sea-water density typically is given as 1.026–1.030 kg^*^L^−1^, and the density of fish usually is ~1.08–1.10 kg^*^L^−1^. To achieve neutral buoyancy, this weight must be balanced by lift. Thus, the lift (L) required is

(1) $$\text{L}={\text{V}}^{*}{\text{g}}^{*}(\rho_{\text{b}}-\rho_{\text{w}}),$$

where V is the volume, g is the gravity force, ρ_b_ is the density of the fish, and ρ_w_ is the density of sea-water. From these considerations it can be calculated that in sea-water a 1 kg fish will achieve neutral buoyancy with a swimbladder volume of ~50 ml.

**Figure 4:**
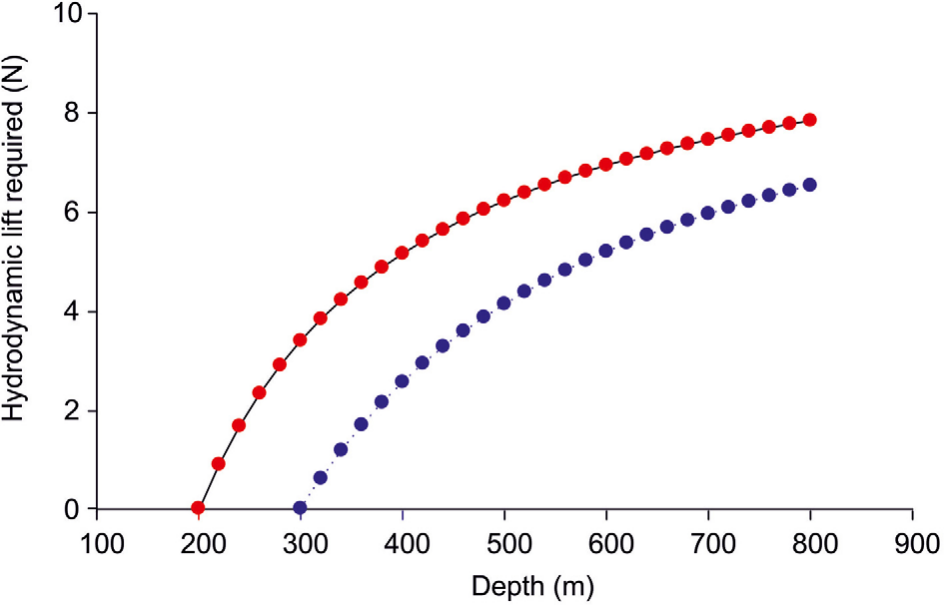
**The lift (in N) required for a 1 kg eel when descending from 200 (red) or 300 m (blue) down to 800 m without compensatory gas secretion, i.e., the swimbladder volume decreases according to Boyle's law**.

Swimming will generate hydrodynamic lift, mainly at the pectoral fins, which are used as hydrofoils, but depending on the structure the peduncular keel may also contribute to the generation of lift (Alexander, 1972; Magnuson, 1978; Gee, 1983). The generation of hydrodynamic lift by swimming, however, induces drag, and work must be done against the drag. If we assume that during a vertical migration at the deeper level the swimbladder volume will be too small to provide neutral buoyancy, the fish needs hydrodynamic lift to compensate for this deficit in order to keep his position in the water column. Figure 4 shows the additional lift required by the eel assuming that it descends from the upper level of 200 or 300 m without keeping the swimbladder volume constant, i.e., with decreasing volume of the swimbladder proportional to the increase in hydrostatic pressure at constant temperature. About 6 Newton will be required to compensate for the negative buoyancy a 1 kg fish will encounter at a depth of 800 m, if it moved down from a depth of 300 m, starting with neutral buoyancy.

Assuming hydrodynamic lift is mainly produced at the fins, the work required for this swimming activity can be estimated as:

(2) $$\text{W}=\text{T}*({\text{P}}_{2}-{\text{P}}_{1})*\text{V}*\rho *\text{g}*\text{U}*\text{D}*{\text{P}}_{2}^{-1},$$

where W is the work required if the fish encounters this drag for a certain time (T), P_1_, and P_2_ are the hydrostatic pressures at the two different depth levels, V is volume, ρ is the density, g is gravity force, U is swimming speed, and D is an estimate of the extra drag the eel must suffer to obtain the extra lift (Alexander, 1971).

**Figure 5:**
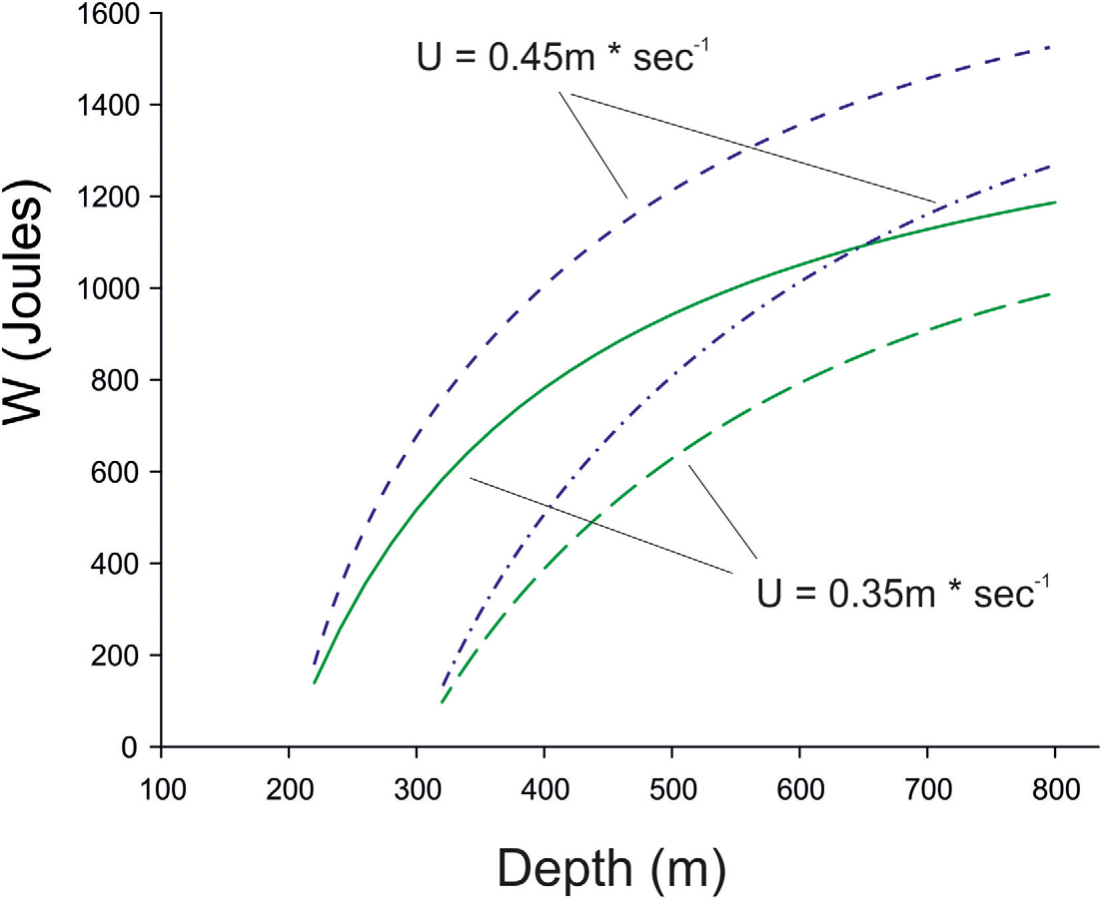
**The work required for a 1 kg eel when descending for 12 h from a depth of 200 or 300 m down to 800 m without compensatory gas secretion, i.e., the swimbladder volume decreases according to Boyle's law**. Swimming speed was assumed to be either 0.35 m^*^s^−1^ (green) or 0.45 m^*^s^−1^, (blue), which is required to reach the Sargasso Sea within a period of 6 month. Long-term swim tunnel experiments have shown that eels with a body length of about 0.8 m can swim for several month at a speed of 0.50 BL^*^s^−1^, which would be equivalent to 0.40 m^*^s^−1^ (Van den Thillart et al., 2004; van Ginneken et al., 2005).

For the extra drag a fish must suffer to obtain the extra lift Alexander (1971) assumed a value of 0.2. If we take this value, which is dependent on the Reynolds numbers of the fins (Alexander, 1972), a swimming speed of 0.35–0.45 m^*^s^−1^, which is required to reach the Sargasso Sea in time (see below) we can calculate the work required during the time period of 12 h for the descent and the stay at the lower depth. The results of this calculation are shown in Figure 5, assuming that a 1 kg eel starts to move down from a depth of either 200 or 300 m with a swimbladder volume of 50 ml and neutral buoyancy, and the decreasing swimbladder volume is not compensated by gas secretion. Depending on the swimming speed the descent down to 800 m will require about 1.0–1.4 kJ for a 1 kg fish within 12 h.

This calculation was originally developed for a “typical” fish, and the elongated eel body looks quite different. Eels do not have a peduncular keel, and this structure therefore cannot contribute to the generation of lift, but the body itself and undulatory movements may influence the generation of hydrodynamic lift (Magnuson, 1970, 1978), but also the level of drag encountered during swimming. Accordingly, the actual values for the eel may slightly deviate from those calculated here, but it is obvious that a significant amount of work must be spent for hydrodynamic lift generation, and this work increases with increasing distance between the upper and the lower level of the diurnal migration.

While this consideration assumes that the fish swim in order to stay at a certain water depth, eels must swim anyway in order to reach their spawning ground. Tracking experiments following 11 silver eels in the North Sea revealed a swimming speed of 0.69–0.96 cm^*^s^−1^, equivalent to 0.6–0.9 Bl^*^s^−1^ (Tesch, 1974; Beamish, 1978). Using long-term swimming experiments the spawning migration was imitated in the lab and silver eels were swum for 3 or for 6 month in a swim tunnel (Van den Thillart et al., 2004; van Ginneken et al., 2005). The swimming speed was set to 0.39 m^*^s^−1^ and to 0.36 m^*^s^−1^ (= 0.5 Bl^*^s^−1^), so that the eels would cover a distance of 5.500 km within 6 month. These experiments demonstrated that eels are able to cover the distance from the European coast to the Sargasso Sea with their on board energy reserves without food intake, and based on the oxygen consumption and on bomb-calorimetry an energy consumption of about 0.42–0.83 kJ^*^kg^−1^^*^km^−1^ was calculated. This swimming speed results in a daily migration of about 30–35 km, accordingly within 12 h the energy consumption amounts to 6.3–14.5 kJ for a 1 kg eel swimming under atmospheric pressure with a status of neutral buoyancy. Accordingly, the energy required to compensate for the decrease in buoyancy during the decent amounts to about 22% of the energy required for swimming with neutral buoyancy in the worst case, and 7% as a minimum estimate. If we assume that in the upper depth range (200–300 m) eels swim more or less in a status of neutral buoyancy the required energy expenditure should be comparable to the values obtained in the swim tunnel experiment.

As already mentioned these calculations are based on a number of assumptions and therefore must be taken as estimates. For the eel in particular it appears possible that the actual energy expenditure required for swimming is lower than calculated, and at depth it may also be lower than measured in the swim tunnel experiments. Swimming performance and slow muscle power output have been shown to be improved in silver eels as compared to yellow eels (Ellerby et al., 2001; Quintella et al., 2010), and it could be that in the *in vivo* situation in migrating eels this improvement is even enhanced. Furthermore, silver eels under pressure swim more efficient than under atmospheric pressure: Oxygen consumption of male silver eels measured at swimming speeds between 0.2 and 1.0 Bl^*^s^−1^ at a pressure of 1 atm and then at 101 atm was significantly lower at high pressure (Sebert et al., 2009a). If this can be transferred to the *in vivo* situation of migrating eels the actual amount of energy required for swimming would be lower than measured in the swim tunnel experiments (Van den Thillart et al., 2004; van Ginneken et al., 2005). This would increase the relative amount of energy required for buoyancy compensation during vertical migrations, but it is unknown how the repeated changes in hydrostatic pressure affect the energy requirements for swimming activity. It therefore would very interesting and important to get a better insight into the swimming efficiency of eels during their spawning migration and on the effect of the repeatedly changing hydrostatic pressure on swimming performance.

Vertical migrations cause a change in hydrostatic pressure and therefore affect the buoyancy status of fish with a compressible swimbladder, but they also increase the distance covered by the fish. From the traces recorded in migrating eels it can be estimated that the descent from 200 to 300 m down to 700–800 m takes about 1.5 to 2.5 h, and ascending to the higher level during the night takes about the same time (Aarestrup et al., 2009). If the change in depth would be achieved by vertical movements, a change in depth of 500 m would increase the distance by 1 km per day. Based on the distance between the European coast and the Sargasso Sea and the time required to cover this distance it can be calculated that eels must swim about 30–35 km per day. If eels would travel at their optimal swimming speed of 0.61–0.68 m^*^s^−1^ (= 0.74–1.02 Bl^*^s^−1^) with a minimum energy expenditure (Palstra et al., 2008) they would even swim more than 40 km per day and reach the Sargasso Sea in less than 5 month. This means, that the vertical migration can at most increase the distance to be covered every day by about 3%, and because eels do not swim vertically it is far less than 3% and therefore probably negligible.

Nevertheless, the vertical migrations increase the energy required for the spawning migration and the question remains, why they are performed. Aarestrup et al. (2009) hypothesized that the vertical migrations are connected to thermoregulatory behavior. The descent to cooler water was supposed to keep average temperature below 11°C, delaying gonadal development, which is only completed towards the end of the spawning migration. Overall metabolic rate also decreases with decreasing temperature, thus the descent may help to reduce energy expenditure. The overall temperature differences between the upper and the lower water level, however, was only slightly above 1°C (Aarestrup et al., 2009), so that other effects may play a role. Most likely predator avoidance may contribute to the daytime descent (Schabetsberger et al., 2013). Studies using popup satellite tags reveal that there is predation on migrating European and American eels, not only near the coast, but also in the open ocean (Béguer-Pon et al., 2012; Westerberg et al., 2014).

## ROS

Because oxygen makes up a large fraction of the swimbladder gas at depth, the high hydrostatic pressures encountered during the vertical migrations must result in very high oxygen partial pressures (several ten or may be hundred atmospheres) in the swimbladder and thus in gas gland cells (Fänge, 1983; Kobayashi et al., 1990b; Pelster, 2009). This leads to another interesting question and important topic: How does the gas gland tissue protect itself from oxygen damage caused by ROS, which are typically generated at high oxygen tensions (Brueckl et al., 2006; Valko et al., 2007; Alfadda and Sallam, 2012)? In the mammalian lung hyperoxic ventilation causes production of O^−^_2_ and H_2_O_2_, which for example, activate endothelial cells contributing to lung injury, and stimulate inflammatory reactions (Chabot et al., 1998; Brueckl et al., 2006). Mitochondria, the main production site for ROS, are not numerous but present in gas gland cells (Dorn, 1961; Pelster, 1995), and the membrane bound enzyme NADPH oxidase (nicotinamide adenine dinucleotide phosphate-oxidase) may also generate superoxide and thus contribute to ROS production. At a PO_2_ of many atmospheres the generation of ROS therefore must be expected.

Our preliminary studies revealed the presence of glutathione reductase activity in gas gland cells of the European eel, while it was not detected in other tissues, and also superoxide dismutase and catalase activity were found in homogenates of eel gas gland tissue, and these enzymes are important for the degradation of ROS (Schneebauer and Pelster, unpublished results). In several marine species activities of these enzymes in swimbladder tissue appear to be higher than in other tissues, but the activity was not correlated to an inflation or deflation of the swimbladder (Morris and Albright, 1981, 1984). The activity of the pentose phosphate shunt, which is important for the CO_2_ production of gas gland cells (see above), can also be seen in this context. In this shunt NADPH+H^+^ is generated, which is used by radical oxidizing enzymes like glutathione reductase for the detoxification of ROS. While eels in freshwater with a limited water depth probably hardly experience an oxygen partial pressure of more than a few atmospheres, during the spawning migration the expected oxygen partial pressures are many times higher. Accordingly, it can be expected that the silvering process includes a significant increase in the capacity of the swimbladder tissue to deal with ROS in order to avoid tissue damage. A detailed analysis of the oxygen defense systems in silver eels therefore appears to be quite promising and may provide interesting insights into the mechanisms preventing tissue damage due to ROS production, or, alternatively, elucidate how excessive ROS production can be prevented in the presence of high oxygen partial pressures.

## Anguillicola

**Figure 6:**
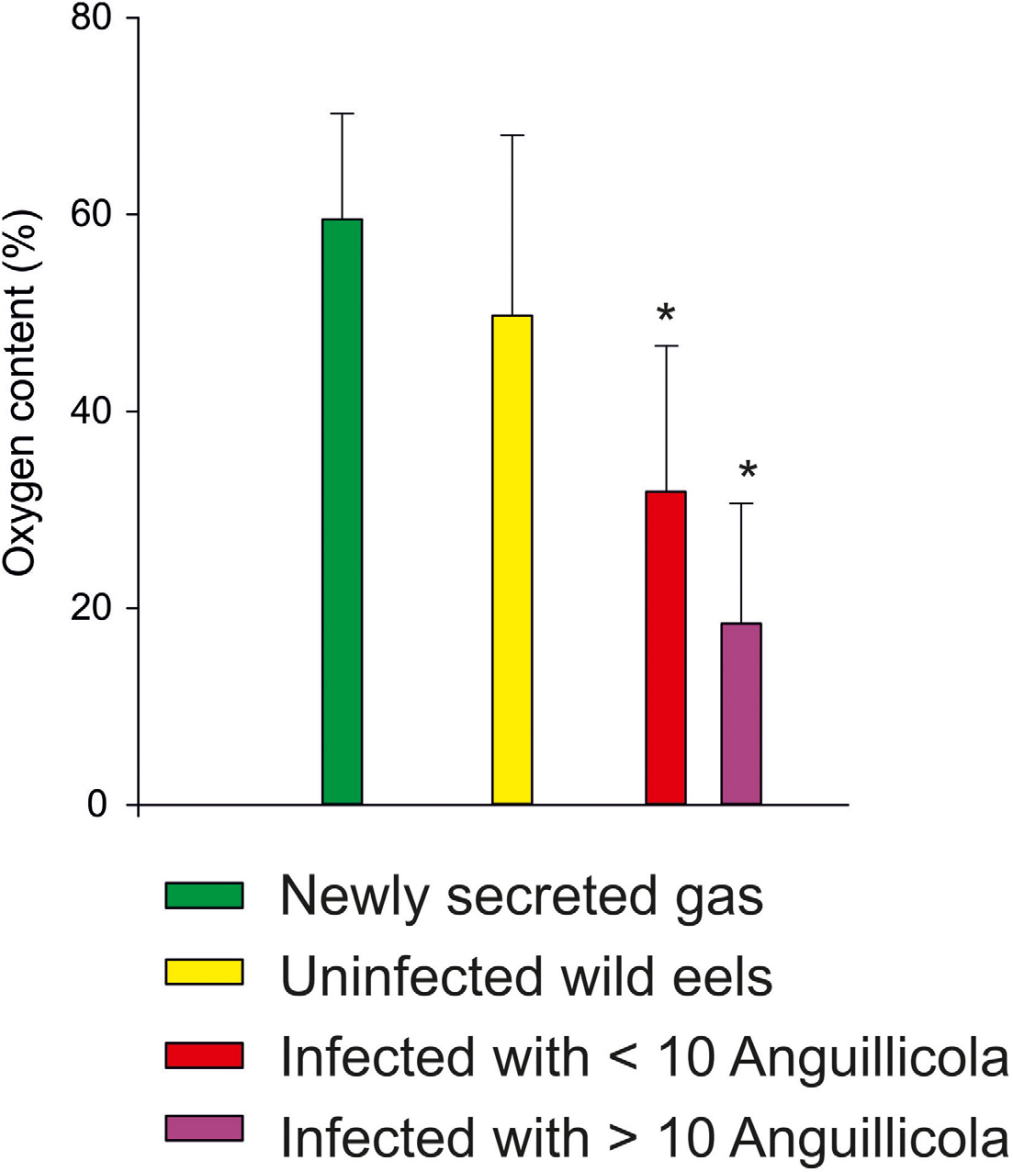
**Swimbladder oxygen content measured in lab experiments (green, Kobayashi et al., 1990b) and in swimbladder gas samples taken from wild eels not infected with *Anguillicola crassus*, and from eels infected or heavily infected with the nematode (data from Würtz et al., 1996)**. Mean ± S.D.; ^*^ indicates significant differences between uninfected and infected wild eels, *p* < 0.05, Kruskall–Wallis-test.

The recent decline in the population of the European eel may in part be related to an infection of the swimbladder with the nematode parasite *Anguillicola crassus* (= *Anguillicoloides crassus*). This sanguivorous, histotrophic nematode was brought to Europe in the 1980's and within a decade a large fraction of the European eels was infected (Moravec, 1992; Schabuss et al., 2005). An infection of the swimbladder results in severe alterations of the swimbladder epithelium (Nimeth et al., 2000; Würtz and Taraschewski, 2000), and based on the macroscopical appearance of the swimbladder and the exudates present in the bladder an infection dependent degeneration of the swimbladder was recently confirmed (Lefebvre et al., 2013). Given the importance of gas gland cell metabolism for the initiation of gas secretion these histological alterations of the swimbladder tissue, which include the formation of a multilayered epithelium with an increase in the diffusion distance between the blood and the swimbladder lumen, was expected to cause an impairment of swimbladder function. A detailed analysis of the swimbladder gas composition and of the rate of gas secretion in relation to the level of infection indeed revealed a significant impairment of swimbladder function by this nematode (Würtz et al., 1996). The rate of gas secretion was significantly reduced in infected eels, and the fraction of oxygen within the newly secreted gas was significantly lower than in uninfected eels (Figure 6), suggesting that the blood acidification by the gas gland cells and the countercurrent concentrating ability was impaired by the nematode. This is in line with the observation that infected eels have a lower number of circulating erythrocytes and thus a reduced oxygen carrying capacity in their blood (Boon et al., 1990).

In addition, swim tunnel experiments showed that infected silver eels have a lower cruising speed, and the costs of transport were elevated by about 20%. Almost 50% of the eels with a heavily infected swimbladder stopped swimming at comparatively low swimming speeds, and in a long term swimming experiment mimicking the spawning migration infected eels showed an early migration failure (Palstra et al., 2007). Taken together these data demonstrate that the reduced swimbladder function due to an infection with *Anguillicola crassus* impairs swimming performance of the eel and thus increases the energy demand for the spawning journey. A recent study suggested that an artificial infection of eels with the nematode may advance the silvering process (Fazio et al., 2012). Given the thickening of the swimbladder epithelium and the reduction in gas deposition in response to an infection, however, it is expected that in infected eels the silvering related adaptations of gas gland cell physiology cannot occur as to be expected for an uninfected swimbladder, and the elasticity of the swimbladder wall of infected eels has been shown to be significantly reduced as compared to uninfected eels (Barry et al., 2014). The results so far clearly suggest that the infection with the nematode will impair the spawning migration to the Sargasso Sea, and as a worst case scenario it might even make a successful spawning migration impossible.

## Perspectives

The fish swimbladder and in particular the eel swimbladder and its role during the spawning migration has fascinated scientist for more than a hundred years, but it still remains a mystery. Recent molecular studies and experiments trying to artificially induce silvering provided significant insight and indicate that silvering is more like the onset of puberty than a genuine metamorphosis (Aroua et al., 2005). The molecular changes in gas gland tissue, however, associated with the process of silvering have not been analyzed so far. Given the importance of the swimbladder during the spawning migration this appears to be a promising area, and first studies are underway. In this context it also would be interesting to see how the tissue is able to avoid damage caused by ROS or alternatively, how the tissue is able to avoid the generation of ROS in the presence of hyperbaric oxygen pressures.

While the first swim tunnel experiments have been performed under constant pressure and therefore in a status of neutral buoyancy, in order to better understand the spawning migration it would be necessary to analyze the swimming performance and energetics under conditions of variable negative buoyance as to be expected in the migrating eel. Although the first swim tunnel experiments suggest that the swimbladder nematode *Anguillicola crassus* impairs swimming performance, it is still unclear whether eels with an infection or eels suffering from a previous infection will be able to successfully migrate to the Sargasso Sea, and how the infection affects the silvering process in the swimbladder. Our knowledge about swimbladder function in the eel and the spawning migration has been significantly advanced over the last hundred years, but many questions remain. Answers to these questions are necessary to better understand population dynamics of the European eel and to be able to find appropriate means to stabilize the currently declining populations.

### Conflict of interest statement

The author declares that the research was conducted in the absence of any commercial or financial relationships that could be construed as a potential conflict of interest.
